# Snap‐On PMMA Provisional Restorations in a Full Digital Workflow for the Rehabilitation of Worn Dentition: A Clinical Report

**DOI:** 10.1111/jerd.70040

**Published:** 2025-09-28

**Authors:** Theodoros Tasopoulos, Panagiotis Zoidis, Heidar Shahin, George Kouveliotis, Vassiliki Rizou, Olga Naka

**Affiliations:** ^1^ Private Practice Athens Greece; ^2^ Department of RDS, Division of Prosthodontics University of Florida College of Dentistry Gainesville Florida USA; ^3^ Master Dental Technician Athens Greece; ^4^ Department of Prosthodontics, School of Dentistry Aristotle University of Thessaloniki Thessaloniki Greece

**Keywords:** digital workflow, full‐arch rehabilitation, milled provisional restorations, snap‐on PMMA, tooth wear

## Abstract

**Objective:**

To highlight the application of digitally fabricated Snap‐On PMMA provisional restorations as a minimally invasive and adaptable methodology for assessing the vertical dimension of occlusion and occlusal stability during the comprehensive rehabilitation of worn dentition.

**Clinical Considerations:**

A clinical case is presented involving a 58‐year‐old male with severe anterior tooth wear, multiple edentulous spaces, and occlusal dysfunction. The patient underwent full‐arch digital rehabilitation through a workflow incorporating intraoral scanning, facial scanning, cone‐beam computed tomography (CBCT), and virtual smile design. Snap‐On PMMA milled provisional restorations enabled real‐time evaluation of the proposed VDO, phonetics, esthetics, and centric occlusion in a reversible, non‐invasive manner. Their use also enabled immediate implant loading at site #22 and facilitated the digital transfer of the validated occlusal scheme from the provisional stage to the definitive CAD/CAM restorations. Definitive restorations were fabricated using monolithic zirconia and lithium disilicate, selected for their superior mechanical strength, longevity, and esthetic potential.

**Conclusion:**

Snap‐On PMMA provisional restorations represent an effective diagnostic and transitional tool in the management of worn dentition. Integrated into a fully digital workflow, they enhance treatment predictability by allowing reversible clinical validation and direct digital transfer of occlusal parameters to the final prostheses. Combined with advanced restorative materials, this approach improves treatment predictability, patient satisfaction, and the long‐term clinical success of managing complex cases of worn dentition.

**Clinical Significance:**

Snap‐On PMMA provisionals offer a minimally invasive, reversible way to assess occlusion, function, and esthetics before final restoration. This technique enhances accuracy and predictability in full‐mouth rehabilitations, making it highly valuable in esthetic dentistry.

## 1 Introduction

Tooth wear is characterized by the progressive loss of tooth structure, primarily driven by the mechanisms of attrition, abrasion, and erosion, which frequently interact in synergy [1, 2]. It is a notable occurrence that tends to advance with the aging process [3]. However, several factors should be considered concerning the reasons for excessive tooth wear. These factors include coarse dietary habits, stress‐related conditions such as bruxism, and psychological disorders like bulimia, which can also contribute to the condition [1, 2]. Additionally, medical conditions, including gastroesophageal disorders and obstructive sleep apnea, have been shown to exacerbate the condition [1, 2, 3, 4, 5]. Identifying all contributing factors is essential to facilitate comprehensive treatment planning.

The diagnosis of worn dentition presents significant challenges, attributable to its multifactorial nature. Therefore, a meticulous analysis of patient data is essential, encompassing a detailed medical and dental history, an assessment of dietary habits, and a comprehensive clinical and radiographic examination. A thorough clinical assessment should include both extraoral and intraoral evaluations, along with considerations of esthetics, phonetics, and functional performance [5, 6].

The increasing demand for esthetic rehabilitation underscores a significant patient inclination toward enhancing their physical appearance [5]. Meticulous protocols for managing worn dentition require a thorough assessment, coupled with controlled modification of the vertical dimension of occlusion (VDO) to restore both occlusal stability and functional efficiency.

The use of digitally milled snap‐on PMMA provisional restorations within a fully digital workflow offers a minimally invasive and reversible method for testing the proposed VDO, esthetics, phonetics, and occlusal scheme before final treatment [7]. Unlike conventional bis‐acrylic or manually fabricated temporaries, milled PMMA provisionals demonstrate superior mechanical and optical properties, including higher flexural strength, greater wear resistance, and improved polishability. These qualities enhance biocompatibility with adjacent soft tissues [8, 9, 10, 11, 12]. Additionally, the digital design file for the provisional can be stored and reused if necessary, providing efficiency and reliability throughout the rehabilitative process [7]. Recent literature highlights these provisionals' clinical value as diagnostic and transitional tools in comprehensive rehabilitation [7, 13, 14]. Overall, these findings establish milled PMMA provisionals as a state‐of‐the‐art solution that combines excellent material properties with the flexibility of a snap‐on design, making them particularly suitable for managing complex cases of worn dentition within a fully digital workflow.

Literature suggests that the preferred treatment protocols typically comprise extensive elective endodontic therapy alongside comprehensive full‐crown coverage across most dentitions [6]. Contemporary minimally invasive techniques, such as adhesive restorations and biomimetic approaches, emphasize the preservation of sound tooth structure while optimizing biomechanical performance [15, 16]. Furthermore, integrating high‐performance restorative materials, including lithium disilicate and zirconia‐based ceramics, improves longevity, wear resistance, and predictability under functional loads. These advanced techniques effectively address the structural and esthetic deficiencies associated with dental wear [5, 7].

The digital workflow implemented in rehabilitating worn dentition aligns with the standardized protocols of contemporary reconstructive dentistry, integrating precision‐driven methodologies that enhance treatment predictability and efficiency. This workflow encompasses three pivotal phases: *Data acquisition*, which employs intraoral scanning, extraoral 3D facial scanning, and cone beam computed tomography (CBCT) for comprehensive capture of hard and soft tissue morphology; *Data processing and treatment planning*, which involves virtual articulation, digital wax‐ups, and AI‐assisted occlusal analysis to optimize both functional and esthetic outcomes; and *treatment execution and prosthesis fabrication*, incorporating computer‐aided design/computer‐aided manufacturing (CAD/CAM), 3D printing, and additive/subtractive manufacturing technologies to ensure precise restorative adaptation and long‐term durability. Integrating artificial intelligence, machine learning, and virtual patient simulation further enhances treatment accuracy and efficiency, providing a predictable approach to managing worn dentition with minimal invasiveness [14, 17, 18, 19, 20, 21].

## 2 Case Presentation

**FIGURE 1:**
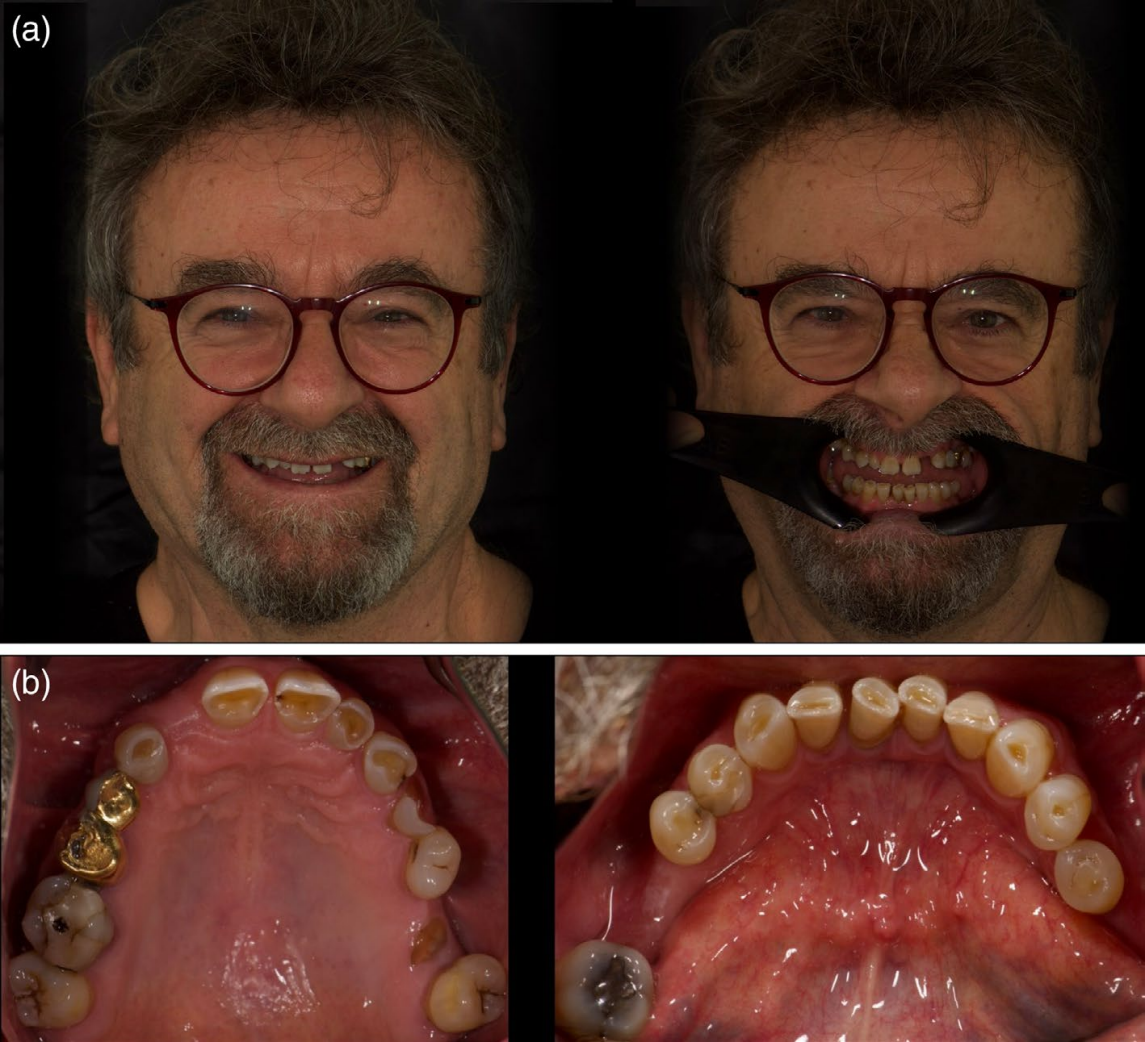
(a) Pre‐operative patient's frontal smile view. (b) Pre‐operative occlusal view demonstrating generalized tooth wear and edentulous sites (ACE Class IV classification).

A 58‐year‐old male patient with no significant medical history presented with extensive fractures involving the anterior dentition. His primary concerns were challenges associated with mastication and a deficiency in self‐assurance regarding his ability to smile. Clinical evaluation revealed pronounced wear of the anterior dentition, characterized by a reduction in clinical crown height, which resulted in a compromised esthetic outcome and a deficiency in anterior guidance. Clinical history revealed that acid erosion was aggravated by parafunctional attrition and abrasion. In addition, multiple single missing teeth were documented in the upper and lower arches. Moreover, a tooth fracture and a residual root were noted in the posterior region of the left maxilla (Figure 1a,b). The CBCT analysis of the temporomandibular joints (TMJ) demonstrated a subtle anterior displacement and bilateral dislocation of the condyles in maximum intercuspation (MIC). Notably, a decreased distance was observed between the anterior aspect of the condyle and the inferior surface of the anterior wall of the glenoid cavity.

The transformation of patient information into digital data began with data acquisition, a critical step that lays the groundwork for subsequent procedures, including analysis, treatment planning, and data processing. This phase utilized advanced technologies, including digital photographs, intraoral scanners, smile design software, and cone beam computed tomography (CBCT).

**FIGURE 2:**
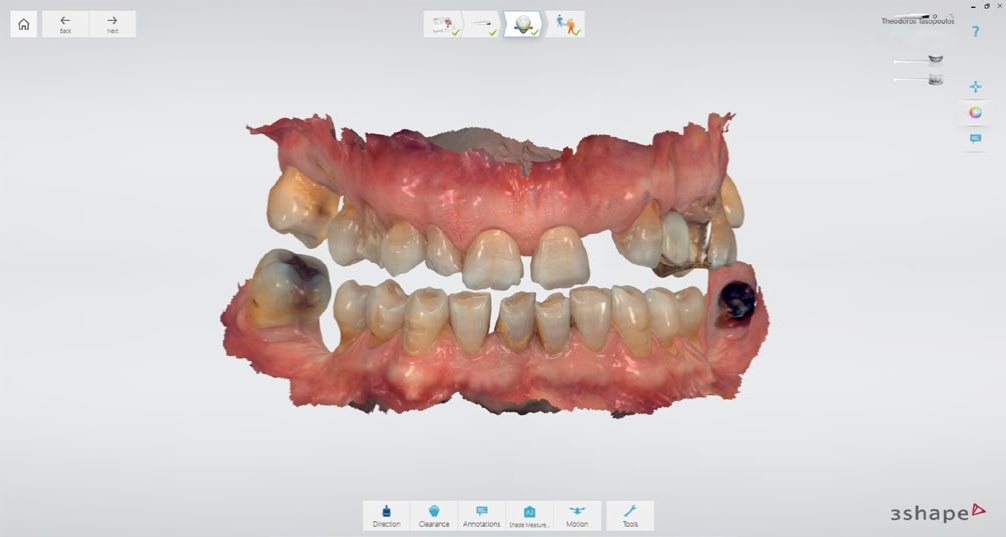
Pre‐operative intraoral scans recorded at the desired OVD in CR, using an anterior deprogrammer.

**FIGURE 3:**
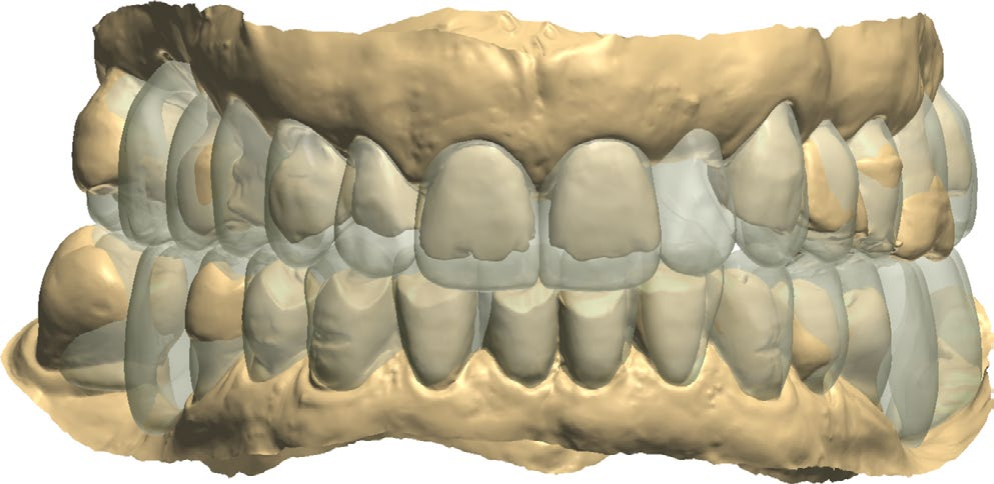
Visualization of VDO alterations using the 3Shape Transparency Tool.

During the initial appointment, intraoral scans of the upper and lower arches were acquired, and intermaxillary digital registration was conducted at the desired OVD in CR, using a 3D‐printed anterior deprogrammer (Figure 2). An increase in VDO was indicated to enhance the esthetic appearance while simultaneously providing adequate prosthetic space. Figure 3 illustrates the quantitative increase in VDO, assessed with the 3Shape transparency tool, which allows for direct visual comparison between pre‐ and post‐treatment situations.

**FIGURE 4:**
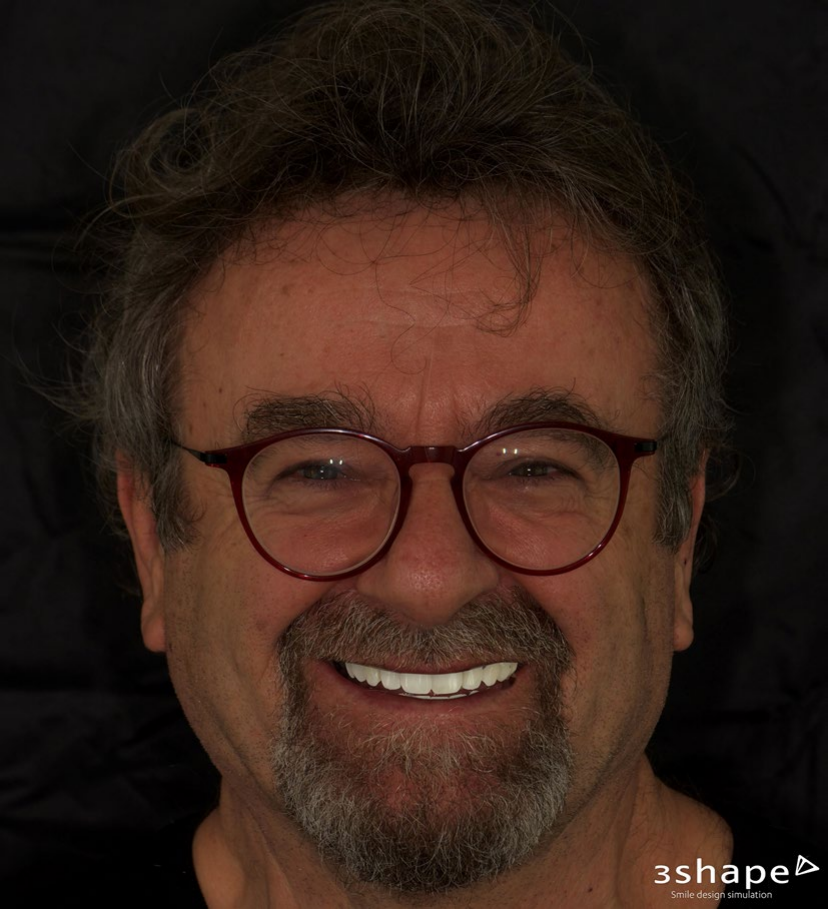
Digital Smile Design treatment simulator.

Following data acquisition, the digital workflow proceeded to the data merging stage. Digital Smile Design (DSD) images from Trios Smile Design (3Shape A/S, Copenhagen, Denmark) (Figure 4) and Standard Tessellation Language (STL) files were superimposed into computer‐aided design (CAD) software (Dental System, 3Shape A/S, Copenhagen, Denmark). This integration facilitated the virtual arrangement of teeth while accounting for the spatial requirements necessary for manipulating VDO.

After thoroughly evaluating the available treatment modalities and patient‐specific factors, the final treatment planning included implant placement in the edentulous areas in both jaws. A contemporary software platform (Implant Studio, 3Shape A/S, Copenhagen, Denmark) facilitated the seamless integration of CBCT data with digital impressions (STL files). This integration allows for the digital superimposition of volumetric CBCT data onto surface scans, providing clinicians with comprehensive three‐dimensional insights into hard and soft tissue anatomy [22]. After virtual implant planning, upper and lower 3D printed fully guided surgical splints were fabricated to accurately transfer the prosthetic plan, ensuring the optimal 3D positioning of the implants at the surgical site. Implants (Paltop PCA, Keystone Dental Group, CA, USA) were strategically placed at the following sites: #36 (5.0 × 6 mm), #37 (4.2 × 6 mm), #46 (4.2 × 10 mm), #16 (4.2 × 11.5 mm), and #24 (3.25 × 10 mm), using a fully guided protocol. Implant site preparation was performed according to the manufacturer's drilling sequence, under copious irrigation at 800 rpm, followed by insertion at a final torque of 40 Ncm. Customized healing abutments (Cervico, VPI, Cyprus) were immediately affixed to the implants to facilitate optimal peri‐implant tissue emergence and to establish the desired gingival margin location. A two‐stage surgical protocol was followed to promote controlled osseointegration.

**FIGURE 5:**
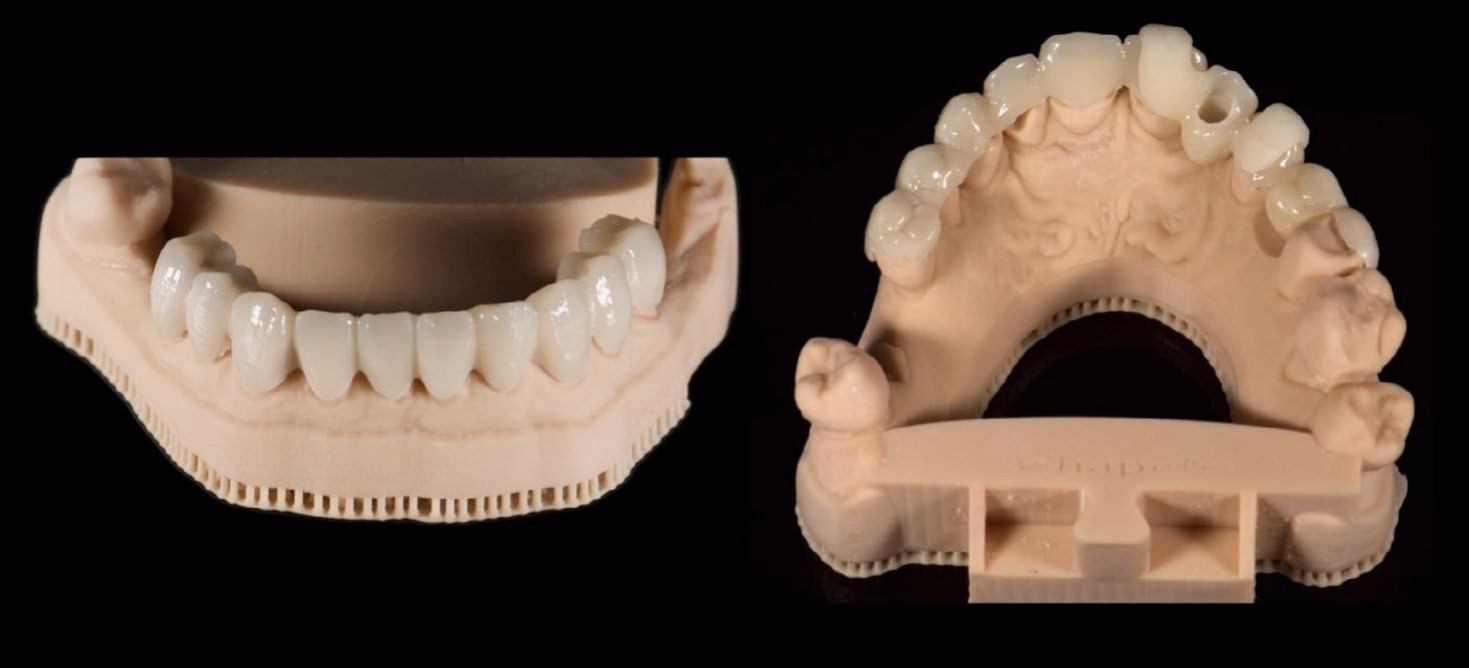
Milled splinted snap‐on temporary PMMA restorations.

In the anterior maxilla, implant #22 (3.8 × 11.5 mm, Paltop PCA, Keystone Dental Group, CA, USA) was placed using a fully guided approach with a final insertion torque of 40 Ncm. Immediately after placement, a screw‐retained provisional crown was fabricated and incorporated into the splinted snap‐on PMMA provisional restoration (Figure 5). Integrating the implant‐supported interim crown within the snap‐on framework provided stabilization, accurate positioning, and controlled functional loading while minimizing the risk of micromovement during the osseointegration period. The snap‐on design further permitted easy removal and reinsertion of the provisional crown for hygiene and monitoring after the osseointegration of the anterior implant. This approach represented a reversible and predictable method of immediate loading within the context of full‐arch rehabilitation, demonstrating the clinical feasibility of combining digital provisionals with immediate implant therapy.

In areas where the diagnostic wax‐up resulted in voids due to the spatial position of the natural teeth (Figure 3), additional material was strategically added to eliminate perforations and ensure a continuous, structurally robust form suitable for the milling process. All observed undercuts were systematically addressed in the preliminary digital design phase. Additionally, the path of insertion was strategically assessed at this stage to optimize seating. The splinted snap‐on milled PMMA restorations were used to evaluate the newly established VDO, the esthetic outcomes, and phonation while simultaneously allowing for monitoring and reprogramming of the patient's dynamic occlusion. The result was a stable centric occlusion characterized by a mutually protected scheme. For refinement, the snap‐on provisionals were rescanned and digitally compared with the original design, enabling minor corrections before finalizing the definitive tooth arrangement.

**FIGURE 6:**
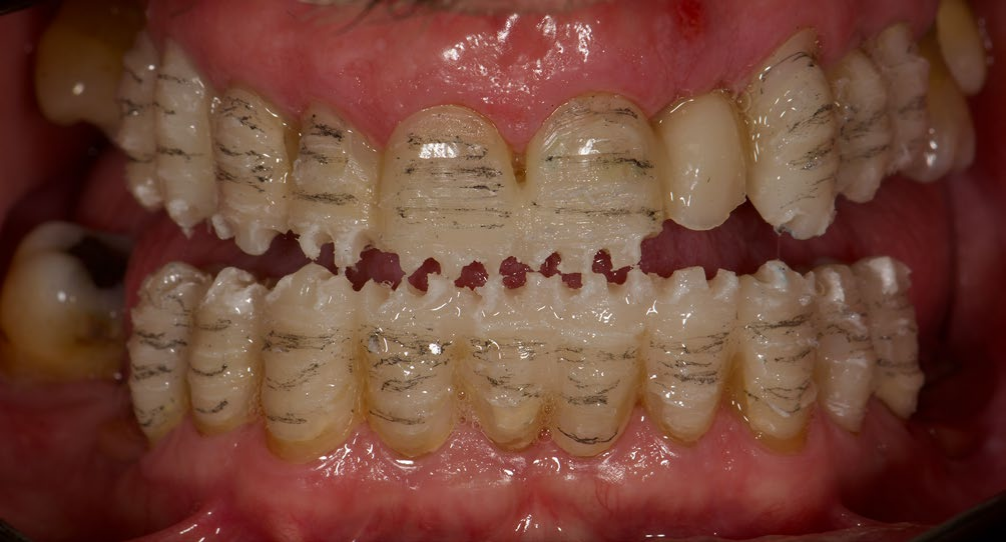
The intraoral mock‐up was approved by the patient and utilized as a reference guide for the conservative preparation depth required for definitive restorations.

**FIGURE 7:**
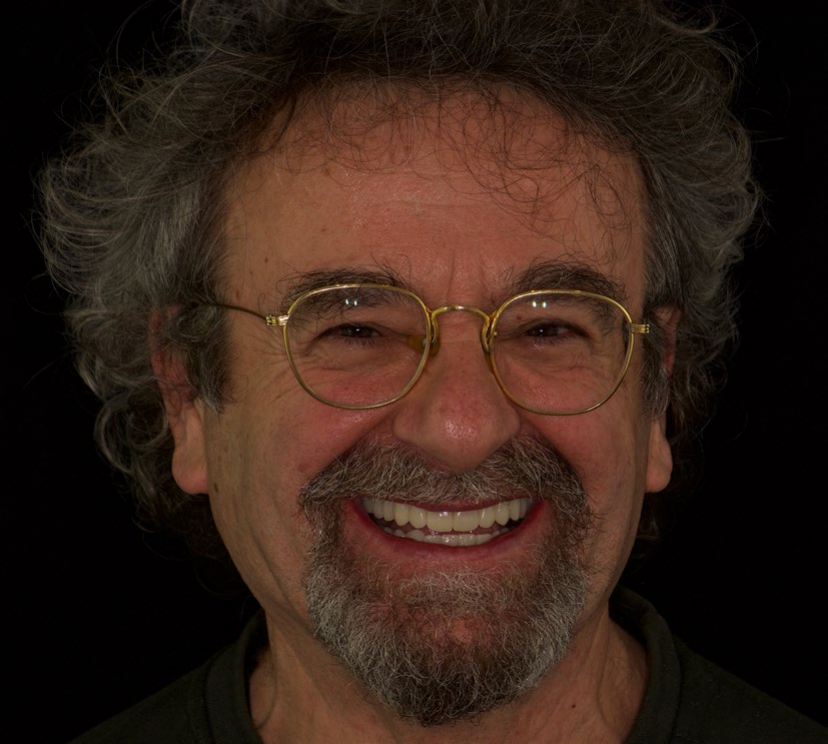
Upper and lower milled PMMA provisionals.

A silicone index was fabricated on a printed cast based on the definitive digital wax‐up to facilitate precise tooth preparations. A mock‐up was made using Bis Acryl. After confirming adequate spatial allowances, minimally invasive preparations were conducted over the mock‐up (Figure 6), establishing a consistent insertion path for the definitive restorations. This process included the refinement of marginal edges. Milled PMMA shells were relined and temporarily cemented (Figure 7).

**FIGURE 8:**
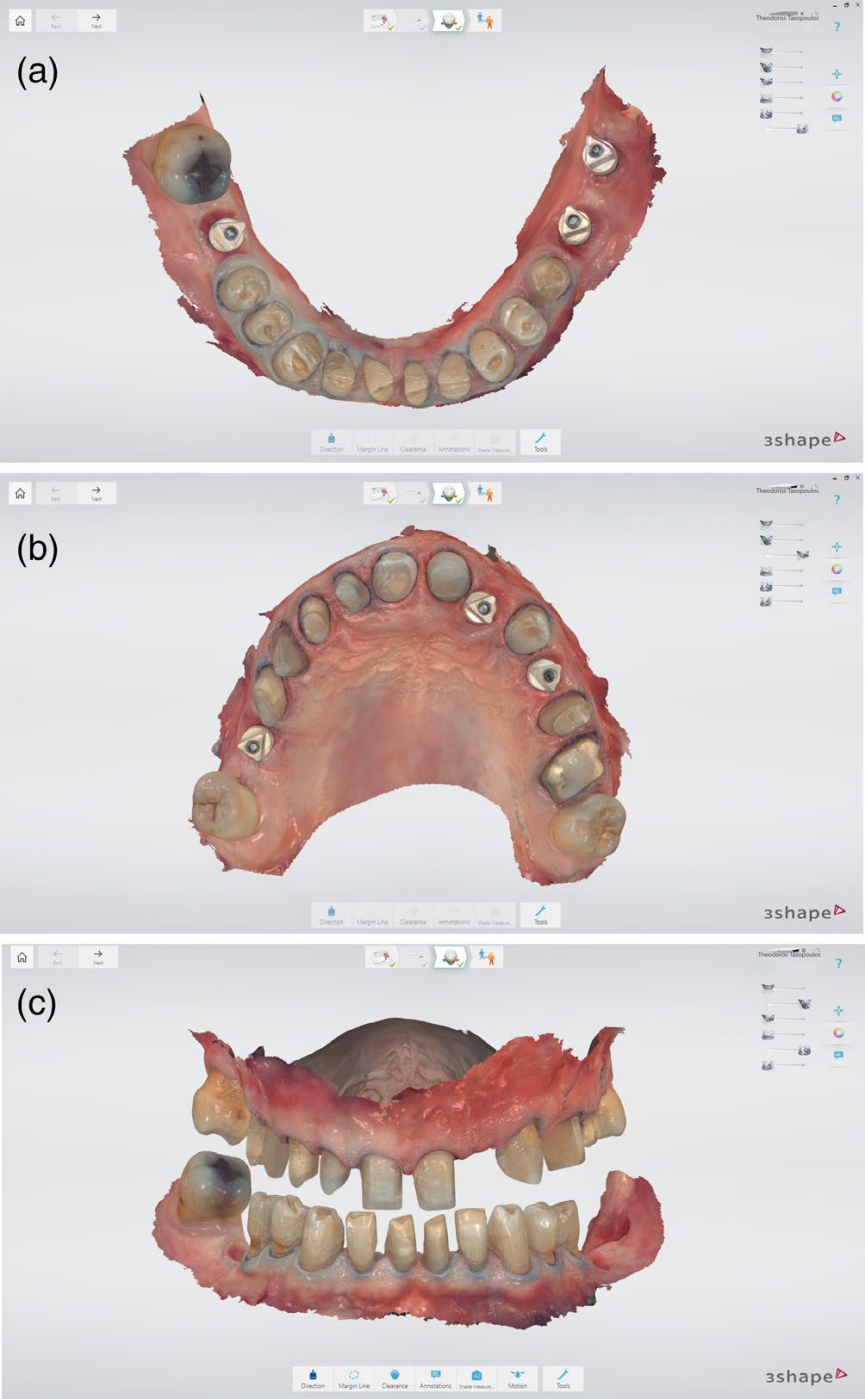
(a–c) Master digital impressions. Intermaxillary digital registration was performed at the same treatment position.

Upon the completion of the 4‐month post‐surgical healing period, a master digital impression was acquired using the Trios implant strategy. This included a pre‐preparation scan conducted with the individual healing abutments and provisional restorations in situ. After removing both teeth and implant restorations, an emergence profile scan was promptly performed to capture the gingival contours surrounding the implants, thereby preventing the potential collapse of the peri‐implant tissues. A bite registration was obtained based on the posterior bilateral contacts. The preparation scan was obtained using two retraction cords placed around the prepared teeth in conjunction with the positioning of scan bodies to accurately transfer the 3D position of the implants to the laboratory (Figure 8a–c). Additionally, during the impression appointment, the provisional implant‐supported screw‐retained prosthesis was digitally captured in an extraoral manner as an additional scan.

The STL files generated from the digital scanning process were imported into CAD software (Dental System, 3Shape A/S, Copenhagen, Denmark) and merged into a single file. This new file integrated all pertinent data, including the 3D position of the implant, the contours of the prostheses, and the transmucosal profile of the peri‐implant soft tissues. Definitive restorations were designed using the CAD software.

**FIGURE 9:**
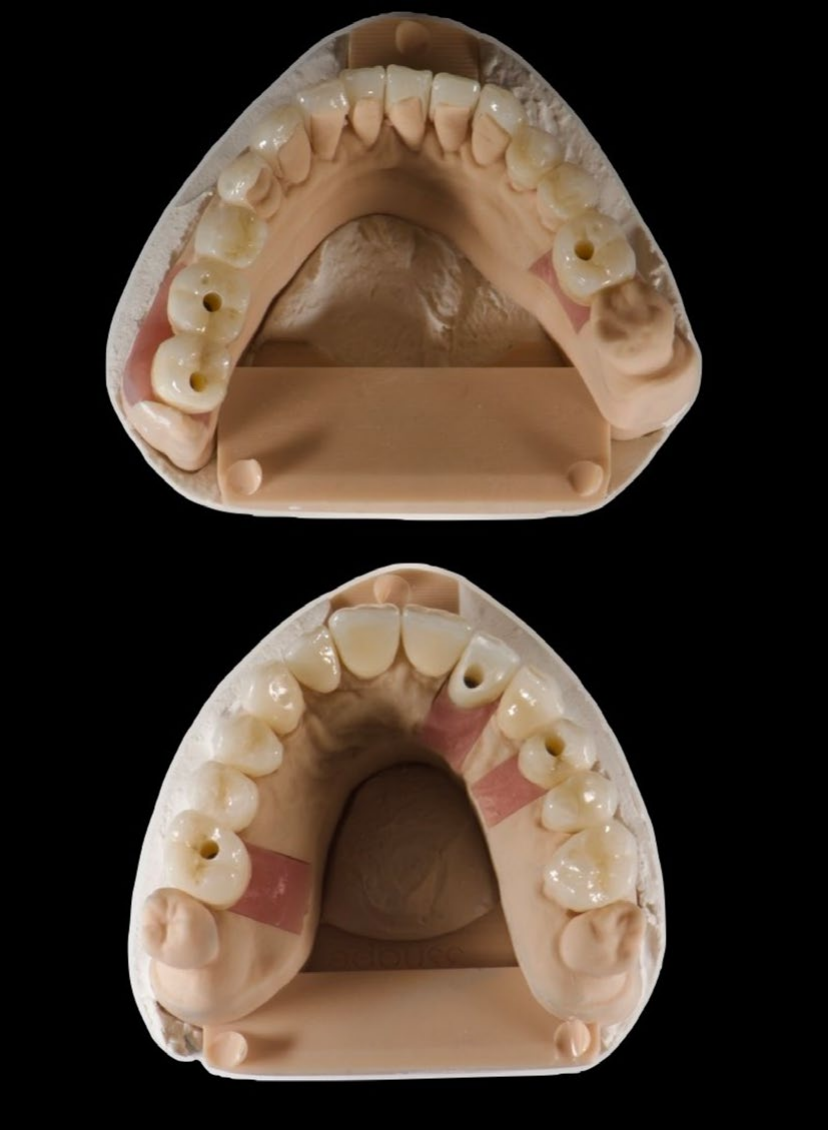
Definite monolithic zirconia restorations.

The definitive fixed dental prostheses (FDPs) were fabricated using a monolithic zirconia‐based material (Figure 9). The final dental restorations underwent thorough testing before being secured with a self‐curing luting composite. Screw‐retained implant crowns were installed and torqued to a specified force of 35 Ncm. To safeguard the screw, a Teflon plug was inserted, and the access hole was covered with composite resin. Minor occlusal adjustments were performed as necessary. Additionally, a Michigan‐type night guard was fabricated and delivered to the patient.

**FIGURE 10:**
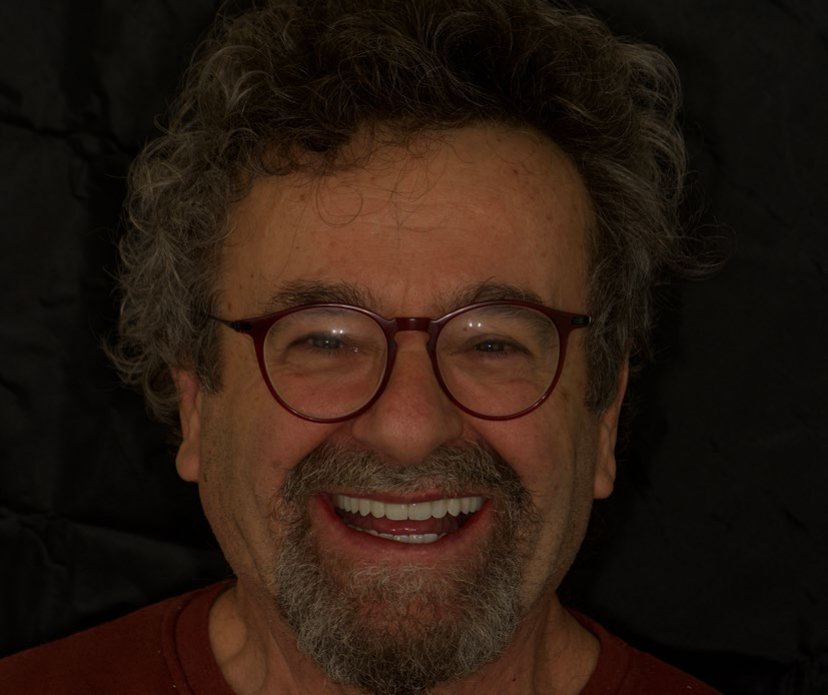
Three months post‐delivery.

The patient reported a high level of satisfaction with the treatment outcome and indicated no complications or complaints following the procedure (Figure 10).

## 3 Discussion

Incorporating digital workflows in dentistry offers multiple advantages over traditional methods, including enhanced communication among the dental team, improved treatment coordination, reduced patient visits, and more predictable clinical outcomes. Nevertheless, their widespread adoption in full‐arch rehabilitation is hindered due to the substantial initial investments required, the prolonged learning curve, and existing technological limitations. Ongoing advancements in scanning accuracy, software integration, and sophisticated manufacturing techniques are anticipated to mitigate these challenges and promote the broader implementation of digital protocols in complex restorative procedures.

Intraoral scanners have demonstrated reliable accuracy compared to conventional impression techniques in specific clinical situations, alongside notable advantages in patient comfort and acceptance [23, 24]. Raja et al. conducted an in vitro comparative analysis of digital versus conventional impressions in fixed prosthodontics. Their findings indicated that digital impressions enhance accuracy and efficiency compared to conventional methods, which may contribute to improved treatment outcomes and patient satisfaction [25]. Another systematic review concluded that various intraoral scanner systems demonstrate high accuracy in the fabrication of inlay, onlay, and veneer restorations. However, variations were observed based on the particular scanner used and the type of restoration. While digital impressions have been shown to offer clinically acceptable precision, a necessity remains for further research aimed at optimizing scanning protocols and improving consistency among different scanning systems [26]. Moreover, a systematic review and meta‐analysis conducted by Papaspyridakos et al. evaluated the accuracy of digital implant impressions obtained using intraoral scanners. This study revealed that IOS impressions present higher accuracy than conventional impressions [27]. Another systematic review and meta‐analysis similarly reported that digital impressions using IOS displayed comparable or superior linear accuracy in relation to conventional impression techniques. However, the accuracy was influenced by several factors, including the scan body type, the specific IOS utilized, the scanning strategy employed, and any modification techniques applied [28].

A review conducted by Chinam et al. discussed the various factors influencing the accuracy of virtual occlusal records acquired via intraoral scanners [29]. The study revealed that discrepancies in occlusal contacts may occur following the alignment of identical virtual casts or occlusal records, contingent on the specific software utilized for processing. Additionally, the analysis identified the presence of stitching errors and a phenomenon referred to as the “tilting effect,” which results from the IOS's orientation of virtual casts relative to the interocclusal record. These factors may adversely affect alignment and consequently lead to distortions in occlusal contacts [29]. Another study demonstrated substantial differences between occlusal contacts recorded using IOS and those captured through articulating paper, indicating that virtual records do not invariably provide an accurate representation of clinical reality [30]. IOS‐generated virtual interocclusal records yield acceptably accurate diagnostic results on average, but significant variability exists in terms of trueness and precision. These findings underscore the necessity for meticulous evaluation of virtual occlusal records to ensure their reliability in mirroring the patient's clinical occlusion [31].

The utilization of a 2D virtual smile design approach is increasingly recognized as a valuable conceptual tool aimed at enhancing communication among clinicians, patients, and dental technicians, thereby improving treatment predictability [32]. However, inherent limitations within the existing 2D virtual smile design methodology—specifically, perspective distortion may result in inaccuracies or discrepancies during the conversion from 2D design to 3D diagnostic waxing [33]. The use of a 3D virtual patient addresses this limitation by facilitating improved communication within the interdisciplinary team and the patient [34]. The accuracy of this alignment is contingent upon the accuracy of both the arch and facial scans, in addition to the efficacy of the integration techniques employed [35]. Furthermore, the superimposition of 2D photographs onto 3D virtual models represents a synthesis of planar and 3D data sets. However, this method lacks critical information regarding the anterior–posterior positioning and inclination of the teeth, thereby rendering such data superimposition a comparatively limited enhancement to communication [36].

Moreover, it is crucial to acknowledge that facial scanners are designed primarily as static tools, which prevents them from effectively capturing dynamic facial movements, including facial expressions. The integration of facial mimics into these scans is not feasible. Consequently, multiple face scans must be executed to enhance the comprehensiveness of the data necessary for CAD procedures, documenting varied positional states such as a resting mouth posture, a social smile, and an extreme smile, with different positions, such as mouth closed, social smile, extreme smile, etc., must include more information for the CAD procedure. Additionally, there is a significant paucity of knowledge concerning the accuracy of face scan data, particularly concerning its alignment and integration with model or intraoral scan data. It is also imperative to contemplate the limitations associated with in vitro studies, as such experimental conditions may not adequately replicate the dynamic nature of real clinical environments, where patient movement is unavoidable [37, 38].

Within this context, occlusal stabilization and establishing an adequate vertical occlusal dimension are critical determinants for optimal musculoskeletal function, playing a fundamental role in comprehensive oral rehabilitation. Moreover, the paradigm of minimally invasive treatment has gained significant traction in the management of worn dentition, primarily due to advancements in restorative materials and adhesive bonding techniques [39]. Veneer and implant restorations have emerged as vital components within the comprehensive full‐mouth rehabilitation strategies framework. To further ensure the long‐term stability of the functional scheme, a Michigan‐type night guard was delivered. This appliance is designed to evenly distribute occlusal loads and protect both tooth‐ and implant‐supported restorations from parafunctional stresses.

The implementation of splinted snap‐on PMMA milled provisional restorations offers distinct advantages over conventional bis‐acrylic provisionals. Their superior flexural strength, reduced wear, and enhanced polishability improve longevity and soft‐tissue compatibility [7, 13, 14]. Unlike their cemented counterparts, the snap‐on design allows for reversibility, facilitating non‐invasive assessments of esthetics, phonetics, and VDO. Importantly, the validated occlusal scheme can be directly transferred to the definitive CAD/CAM restorations, minimizing adjustments and improving efficiency.

An innovative element of this case involved the integration of an immediately loaded implant‐supported crown (#22) into the snap‐on provisional framework. This ensured stable functional loading during the osseointegration phase, demonstrating the versatility of snap‐on provisionals as diagnostic and transitional tools within complex digital workflows.

Contemporary literature consistently identifies lithium disilicate and zirconia‐based ceramics as the predominant materials for the fabrication of ceramic restorations, attributed to their superior mechanical properties, esthetic versatility, and proven long‐term clinical success. Evidence suggests that the survival rates of CAD/CAM‐generated tooth and implant‐supported prostheses are comparable to those of conventionally fabricated prostheses [40]. As previously noted, although the selection of restorative material is primarily dictated by clinician experience and preference, an increasing body of literature advocates for the utilization of monolithic restorations. This shift reflects the enhanced mechanical properties, reduced incidence of chipping, and optimized fabrication processes associated with monolithic ceramic materials. The application of monolithic translucent zirconia in restorative procedures is particularly noteworthy, owing to its remarkable mechanical strength, enhanced fracture toughness, and superior optical properties [41]. Its advantageous wear characteristics, such as minimal material degradation and reduced abrasion of opposing dentition, render it a compelling option for patients prone to parafunctional habits. These attributes collectively contribute to the long‐term clinical success of such materials, reinforcing monolithic translucent zirconia as a reliable and esthetically appealing choice for full‐coverage restorations. Furthermore, it is imperative to avoid intraoral occlusal adjustments of ceramic restorations. This can be facilitated by employing prototypes to evaluate the design of the final restorations, allowing for any necessary modifications prior to the finalization of the design and the manufacturing of the restorations. Should occlusal adjustments be deemed necessary, they should be executed exclusively with fine‐grain diamond burs, followed by a thorough polishing sequence [13, 42].

## 4 Conclusion

Snap‐On PMMA provisional restorations provided a practical and efficient strategy for initially validating the proposed VDO, phonetics, esthetics, and centric occlusion in a reversible and non‐invasive manner. Their use further enabled the incorporation of an immediately loaded implant‐supported crown within the snap‐on framework, demonstrating a novel and versatile application in full‐arch rehabilitation. When integrated with CAD/CAM technologies and contemporary restorative materials, this approach enhances treatment predictability, supports patient satisfaction, and contributes to the long‐term clinical success in managing complex worn dentition cases.

## Disclosure

The authors have nothing to report.

## Conflicts of Interest

The authors declare no conflicts of interest.

## Data Availability

The data that support the findings of this study are available on request from the corresponding author. The data are not publicly available due to privacy or ethical restrictions.
